# Enhancing current guidance for psoriatic arthritis and its comorbidities: recommendations from an expert consensus panel

**DOI:** 10.1093/rheumatology/keae172

**Published:** 2024-03-15

**Authors:** Laura C Coates, Marwan Bukhari, Antoni Chan, Ernest Choy, James Galloway, Nicola Gullick, Alison Kent, Laura Savage, Stefan Siebert, William Tillett, Natasha Wood, Philip G Conaghan

**Affiliations:** Nuffield Department of Orthopaedics, Rheumatology and Musculoskeletal Sciences, University of Oxford, Oxford, UK; Department of Rheumatology, Royal Lancaster Infirmary, Lancaster, UK; University Department of Rheumatology, Royal Berkshire NHS Foundation Trust, Reading, UK; Cardiff Regional Experimental Arthritis Treatment and Evaluation (CREATE) Centre, Cardiff University, Cardiff, UK; Centre for Rheumatic Diseases, King’s College London, London, UK; Rheumatology Department, University Hospitals of Coventry & Warwickshire, Coventry, UK; Department of Rheumatology, Salisbury NHS Foundation Trust, Salisbury, UK; Department of Dermatology, Faculty of Medicine and Health, University of Leeds, Leeds, UK; School of Infection and Immunity, University of Glasgow, Glasgow, UK; Rheumatology Department, Royal National Hospital for Rheumatic Disease, Bath, UK; The Wooda Surgery, Bideford, UK; Leeds Institute of Rheumatic and Musculoskeletal Medicine, University of Leeds and NIHR Leeds Biomedical Research Centre, Leeds, UK

**Keywords:** quality of care, best practice, psoriatic arthritis, psoriasis, care recommendations, comorbidities

## Abstract

**Objectives:**

The existing guidelines for PsA cover many aspects of management. Some gaps remain relating to routine practice application. An expert group aimed to enhance the current guidance and develop recommendations for clinical practice that are complementary to the existing guidelines.

**Methods:**

A steering committee comprising experienced, research-active clinicians in rheumatology, dermatology and primary care agreed on themes and relevant questions. A targeted literature review of PubMed and Embase following a PICO framework was conducted. At a second meeting, recommendations were drafted, and subsequently an extended faculty comprising rheumatologists, dermatologists, primary care clinicians, specialist nurses, allied health professionals, non-clinical academic participants and members of the Brit-PACT patient group, was recruited. Consensus was achieved via an online voting platform at which 75% of respondents agreed in the range of 7–9 on a 9-point scale.

**Results:**

The guidance comprised 34 statements covering four PsA themes. *Diagnosis* focused on strategies for identifying PsA early and referring appropriately, assessment of diagnostic indicators, use of screening tools and use of imaging. *Disease assessment* centred on holistic consideration of disease activity, physical functioning and impact from a patient perspective, and on how to implement shared decision-making. For *comorbidities*, recommendations included specific guidance on high-impact conditions such as depression and obesity. *Management* statements (which excluded extant guidance on pharmacological therapies) recommended multidisciplinary team working, implementation of lifestyle modifications and treat-to-target strategies. Minimizing CS use was recommended where feasible.

**Conclusion:**

The consensus group have made evidence-based best-practice recommendations for the management of PsA to enhance the existing guidelines.

Rheumatology key messages

- This consensus programme aimed to complement existing psoriatic arthritis guidelines with practical, clinically relevant recommendations.
- The recommendations covered psoriatic arthritis diagnosis (screening, imaging) and assessment, incorporating disease impact (including from the patient perspective).
- Management recommendations included a multidisciplinary approach for comorbidities, a treat-to-target strategy, and minimization of use of corticosteroids.

## Introduction

PsA is a chronic inflammatory joint disease occurring in approximately one-quarter of individuals with psoriasis (PsO) [1]. It is highly heterogeneous in its presentation, encompassing a range of musculoskeletal manifestations, including peripheral arthritis, axial inflammation (spondylitis), dactylitis and enthesitis [1]. In addition to progressive joint damage and pain, PsA is associated with extra-articular manifestations such as uveitis and IBD, with comorbidities including metabolic syndrome and cardiovascular disease, and it can adversely affect a patient’s quality of life [1–3].

Recent data emphasize the importance of timely diagnosis, as untreated PsA can lead to irreversible joint damage, which is experienced by approximately half of PsA patients within 2 years of diagnosis [1]. However, many patients experience significant diagnostic delay [4], owing in part to the challenges of differential diagnosis and the lack of validated biomarkers [5, 6]. Following diagnosis, comprehensive assessment should consider arthritis, enthesitis, dactylitis, skin/nail disease and axial involvement, as well as the overall impact on individual patients. Comprehensive evaluation facilitates the selection of appropriate treatments that target specific disease domains and associated comorbidities to reduce morbidity and mortality [2]. To achieve optimal patient care, there is a need for clear and actionable guidance for clinicians on screening and referral (many patients with PsO are managed in primary care or dermatology settings), as well as for optimal management of PsA and its comorbidities.

The existing guidelines, such as those provided by EULAR, the British Society for Rheumatology (BSR), the ACR, the National Psoriasis Foundation (NPF) and the Group for Research and Assessment of Psoriasis and Psoriatic Arthritis (GRAPPA), give comprehensive guidance on the diagnosis and pharmacological management of PsA [1, 7]. Owing to the complexity and heterogeneity of the disease, gaps have been identified relating to the application of guidance in clinical practice, ongoing non-pharmacological management, and quality of care benchmarking, often associated with a lack of evidence.

Consequently, an expert consensus group aimed to develop an evidence- and consensus-based set of recommendations for the management of PsA in clinical practice. A consensus programme was undertaken to define minimum and best quality standards for day-to-day PsA management, adding value to existing recommendations and guidelines, and to provide practical strategies and tools for achieving these quality standards and supporting clinicians, without replacing current guidance.

## Methods

The consensus programme was based on a modified Delphi methodology (Supplementary Fig. S1, available at *Rheumatology* online). A steering committee (SC) was formed of UK clinicians experienced in treating PsA (mean 20.1 years, range 1.5–30) and/or widely published in PsA: nine rheumatologists, one dermatologist, one primary care physician and one specialist nurse.

In an initial meeting held in September 2022, the SC discussed where gaps in current guidelines existed, or where clinicians would benefit from extra support in translating these into clinical practice. Four consensus themes were identified: PsA diagnosis; disease assessment; comorbidities; and management. Management of PsA in this context excluded guidance on pharmacological therapies, which is covered in detail by extant guidelines. Questions were drafted within each theme (15 in total), and a targeted literature review (TLR) was conducted to support and inform responses. Given the aim and context of this programme, certain questions relating to clinical practice and interpretation of the guidance were deemed appropriate to be addressed by the committee’s clinical experience. The TLR was performed within Medline, through PubMed and Embase; 10 725 records were identified, with 174 studies being selected for full-text review following the application of the exclusion criteria (Supplementary Fig. S2, available at *Rheumatology* online).

During further meetings in October and November 2022, the results of the TLR were reviewed and consensus recommendations drafted to address each question. In addition to the recommendations, the SC proposed ‘implications for clinical practice’ statements, practical guidance to further support actionability in day-to-day practice. An extended faculty (EF) of UK PsA-interested clinicians and patients was recruited, comprising rheumatologists, dermatologists, primary care representatives, specialist nurses, allied health professionals, non-clinical academic participants and members of the Brit-PACT patient group. Via an online voting platform, each member of the SC and EF indicated an agreement score for each recommendation on a scale from 1 (strongly disagree) to 9 (strongly agree). For scores lower than 7, voters were requested to provide a written rationale. Patients voted on a selection of recommendations, and lay language was applied to facilitate understanding. Consensus was achieved when 75% of respondents gave scores in the range of 7–9. If consensus was not achieved, a re-vote on the updated recommendation was required. In the early stages of development, the main concept of each ‘implication for clinical practice’ was validated with the EF via their voting responses of ‘Yes’, ‘No’, or ‘Not sure’ to each point; this feedback was used to refine the wording and ensure maximum clinical applicability.

At a final meeting in May 2023, the SC discussed the results of the voting, and the implications for clinical practice were refined to improve relevance and to maximize their use from a clinical perspective.

## Results

### Overview

A total of 34 recommendations were drafted by the SC and put to a vote. The invited EF comprised 40 rheumatologists, 11 dermatologists, 2 primary care professionals, 11 specialist nurses, 9 academic professionals and the Brit-PACT patient advocacy group. Of the invited group, 3 nurses, 1 dermatologist, 6 rheumatologists and 6 patients from the Brit-PACT group, in addition to the 12 SC members, voted on the recommendations (*N* = 27 in total), for an overall participation rate of 29.7%.

**Figure 1.**
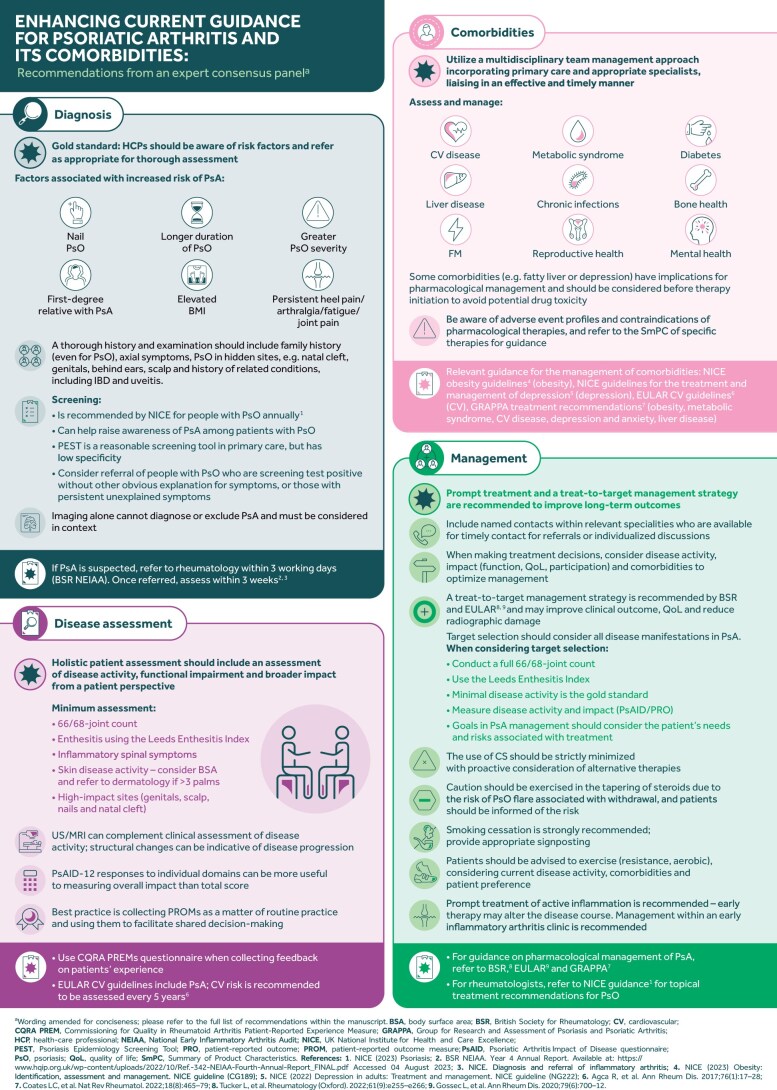
Graphical summary of consensus recommendations

Consensus was achieved for all suggested recommendations, eliminating the need for a second round of voting, with 29 recommendations achieving consensus in the range of 90–100%, 4 in the range of 80–89%, and 1 in the range of 75–79% (Tables 1–4). The questions and recommendations for each theme, and their strength of recommendation and level of consensus are provided below (Tables 1–4), along with the implications for clinical practice (Table 5). A graphical summary of the recommendations and implications for clinical practice is shown in Fig. 1.

**Table 1.** Recommendations, Theme 1: diagnosis

Q1: What factors are associated with a diagnosis of PsA?		
Consensus recommendation	Strength of recommendation^a^	Level of consensus^b^
CR1: Be aware that anyone with PsO or with a family history of PsO may develop PsA.	9 (8.4)	96.3% n/*N* = 26/27
CR2: Be aware that axial disease may be present in a high proportion of PsA patients.	8 (7.5)	85.7% *n*/*N* = 18/21
CR3: When considering a potential diagnosis of PsA, the following factors are associated with increased risk: Nail PsO Longer duration of PsO Greater PsO severity First-degree relative with PsA Elevated BMI	8 (8.1)	95.0% *n*/*N* = 19/20
CR4: Although presentation of PsA may be variable, in people with PsO the following persistent symptoms may warrant consideration of PsA: Heel pain Arthralgia Fatigue Joint pain in a patient with recent onset PsO Enthesitis	8 (8.4)	100% *n*/*N* = 21/21

**Q2. What is the value of PsA screening tools for use in patients with known psoriasis?**		

CR5: Questionnaire-based screening tools have moderate accuracy for screening for PsA, but the cost-effectiveness and number of patients that needs to be screened has yet to be established.	8 (7.4)	81.0% *n*/*N* = 17/21

**Q3. What screening tools should be used/are available in primary care and dermatology?**		

CR6: Patient-completed screening tools may be useful in detecting PsA in patients with PsO, although they have limited specificity.	8 (7.9)	95% *n*/*N* = 19/20
CR7: Be aware that screening tools are not diagnostic tools and cannot prove or exclude a diagnosis of PsA, but they may be useful in determining the need for referral to rheumatology.	8 (8.2)	95.2% *n*/*N* = 20/21
CR8: Consider referral of people with PsO who are screening test positive without other obvious explanation for symptoms, or those with persistent unexplained symptoms.	8 (7.9)	95.2% *n*/*N* = 20/21

**Q4. What diagnostic challenges exist in the identification of PsA? Why are diagnostic delays for PsA so much longer than for RA?**		

CR9: There is a diagnostic delay in patients with PsA compared with RA.	9 (8.2)	89.5% *n*/*N* = 17/19

**Q5. Where and how should imaging be used for PsA diagnosis?** **What features should be assessed in imaging?** **How should non-specialists interpret imaging?**		

CR10: Imaging alone cannot diagnose or exclude PsA and must be considered in context.	9 (8.6)	100% *n*/*N* = 19/19

**Q6. What are appropriate/acceptable timings for referral from primary care to the patient being seen by a specialist?**		

CR11: Aligned with wording used by BSR NEIAA audit: To ensure an accurate and timely diagnosis, adults with suspected persistent joint inflammation (synovitis) in more than one joint, or the small joints of the hands and feet, should be referred to rheumatology services within three working days of presenting in primary care. Once referred, people with suspected persistent joint inflammation should be assessed in a rheumatology service within three weeks.	9 (7.9)	85.7% *n*/*N* = 18/21

a Median score on a 1–9 scale (mean score in brackets).

b Percentage of scores of 7–9 on a 9-point scale. BSR: British Society for Rheumatology; CR: clinical recommendation; NEIAA: National Early Inflammatory Arthritis Audit; PsO: psoriasis.

**Table 2.** Recommendations, Theme 2: disease assessment

Q7: What assessments are most relevant to measure, from the patient perspective?		
Consensus recommendation	Strength of recommendation^a^	Level of consensus^b^
CR12: Best practice for PsA management should involve shared decision-making with alignment of patient and HCP goals.	9 (8.6)	96.3% *n*/*N* = 26/27
CR13: Holistic patient assessment should include an assessment of disease activity, functional impairment and broader impact from a patient perspective.	9 (8.7)	96.3% *n*/*N* = 26/27
CR14: Routine and regular use of patient-reported outcome measures is recommended.	8.5 (8.1)	92.3% *n*/*N* = 24/26
CR15: If auditing quality of care, consider including patient-reported experience measures.	9 (8.3)	100% *n*/*N* = 24/24

**Q8. What are the minimum and best quality standards for day-to-day PsA management in terms of disease assessment?**		

CR16: As a minimum, HCPs caring for someone with PsA should include assessment of joints, enthesitis, spine, skin and comorbidities.	9 (8.6)	100% *n*/*N* = 21/21

**Q9. How should existing imaging be used for ongoing disease assessment and assessing treatment efficacy?**		

CR17: Imaging may be used as an adjunct to support clinical decision-making in terms of whether to change/escalate therapy.	8 (8.3)	100% *n*/*N* = 19/19

a Median score on a 1–9 scale (mean score in brackets).

b Percentage of scores of 7–9 on a 9-point scale. CR: clinical recommendation; HCP: health-care professional.

**Table 3.** Recommendations, Theme 3: comorbidities

Q10: Does coordinated management of comorbidities in patients with PsA improve the likelihood of successful patient outcomes?		
Consensus recommendation	Strength of recommendation^a^	Level of consensus^b^
CR18: Given the limited data on the management of many common comorbidities in the PsA population, we recommend using appropriate condition-specific recommendations to guide management of problems such as hyperlipidaemia, hypertension, diabetes, etc.	9 (8.4)	100% *n*/*N* = 21/21
CR19: Treatment of comorbidities in patients with PsA should utilize a multidisciplinary team management approach incorporating primary care and appropriate specialists in secondary care.	9 (8.4)	96.3% *n*/*N* = 26/27
CR20: In PsA patients who are overweight/obese, a proactive approach to weight loss should be considered following national guidelines and local services.	9 (8.4)	100% *n*/*N* = 20/20
CR21: In PsA patients who are depressed, proactive management should be considered following national guidelines and local services.	8.5 (8.2)	96.2% *n*/*N* = 25/26
CR22: Be aware that some comorbidities (depression, fatty liver disease) have implications for pharmacological management of PsA and should be considered before therapy initiation.	9 (8.6)	95.2 *n*/*N* = 20/21

a Median score on a 1–9 scale (mean score in brackets).

b Percentage of scores of 7–9 on a 9-point scale. CR: clinical recommendation.

**Table 4.** Recommendations, Theme 4: Management

Q11: What are the recommendations regarding use of steroids in patients with PsA?		
Consensus recommendation	Strength of recommendation^a^	Level of consensus^b^
CR23: When making treatment decisions, consider disease activity, impact (function, QoL, participation) and comorbidities to optimize management.	9 (8.5)	95% *n*/*N* = 19/20
CR24: Appropriate multidisciplinary team management (including AHPs) of patients with PsA is recommended for optimal care.	9 (8.7)	100% *n*/*N* = 21/21
CR25: For guidance on pharmacological management of PsA, refer to national and international treatment recommendations.	9 (8.6)	100% *n*/*N* = 19/19
CR26: The use of CSs in PsA should be strictly minimized, with proactive consideration of alternative therapies.	8 (7.4)	75% *n*/*N* = 15/20
CR27: Caution should be exercised in the tapering of steroids in people with PsA due to the significant risk of PsO flare associated with steroid withdrawal, and patients should be informed of this risk.	8 (8.0)	94.7% *n*/*N* = 18/19

**Q12: What are the recommendations regarding non-pharmacological management of PsA?**		

CR28: Smoking cessation support is strongly recommended in line with current national guidelines.	9 (8.7)	96% *n*/*N* = 24/25
CR29: Patients with PsA should be advised to undertake muscle strengthening and general aerobic exercise. The exercise activity should take into account current disease activity, comorbidities and patient preference.	9 (8.6)	100% *n*/*N* = 27/27

**Q13: What is the evidence base for early intervention?**		

CR30: Prompt treatment of active inflammation is recommended to improve long-term outcomes. Referral and management within an early inflammatory arthritis clinic is recommended.	9 (8.6)	100% *n*/*N* = 21/21

**Q14: What are the recommendations regarding ‘treating to target’?** **What domains should be measured/monitored when ‘treating to target’ for patients with PsA?**		

CR31: A treat-to-target management strategy is recommended in line with national and international recommendations.	9 (8.5)	100% n/*N* = 24/24
CR32: Target selection should consider all disease manifestations in PsA. Minimal disease activity is the evidence-based multi-domain target for treatment in PsA.	9 (8.5)	100% *n*/*N* = 24/24
CR33: There should be shared decision-making and alignment of patient and physician goals when discussing treatment options.	9 (8.7)	96.3% *n*/*N* = 26/27

**Q15: What does ‘good’ look like with regard to working with other specialities in the management of PsA?** **How should this be achieved in practice?** **How should extra-articular manifestations be managed?**		

CR34: Collaborative working across key specialities (dermatology, gastroenterology, ophthalmology) is recommended to optimize outcomes for people with PsA; multidisciplinary clinics are recommended.	9 (8.4)	90.5% *n*/*N* = 19/21

a Median score on a 1–9 scale (mean score in brackets).

b Percentage of scores of 7–9 on a 9-point scale. AHP: allied health professional; CR: clinical recommendation; PsO: psoriasis; QoL: quality of life.

**Table 5.** Implications for clinical practice, Themes 1–4

**Theme 1: Diagnosis**
**Statements** **CR1: Be aware that anyone with PsO or with a family history of PsO may develop PsA.** **CR2: Be aware that axial disease may be present in a high proportion of PsA patients.**
**Implication for clinical practice**
When considering a potential diagnosis of PsA, the following factors are associated with increased risk: Nail PsO Longer duration of PsO Greater PsO severity First-degree relative with PsA Elevated BMI A thorough history and examination should include: Family history Axial symptoms PsO in hidden sites, e.g. natal cleft, genitals, behind ears, scalp History of related conditions, including IBD and uveitis
**Statements** **CR5: Questionnaire-based screening tools have moderate accuracy for screening for PsA, but the cost-effectiveness and number of patients who need to be screened has yet to be established.** **CR6: Patient-completed screening tools may be useful in detecting PsA in patients with PsO, although they have limited specificity.**
NICE recommends an annual assessment for PsA in people with PsO. PEST is the most widely used screening tool and is quick to administer. For FCPs seeing patients with MSK in primary care, PEST is a reasonable screening tool, although it should be recognized that this has low specificity.
**Statements** **CR7: Be aware that screening tools are not diagnostic tools, and cannot prove or exclude a diagnosis of PsA, but may be useful in determining the need for referral to rheumatology.** **CR8: Consider referral of people with PsO who are screening test positive without other obvious explanation for symptoms, or those with persistent unexplained symptoms.**
Thorough assessment by a rheumatologist (incorporating clinical, laboratory and imaging factors combined with context) is the gold standard for making a diagnosis. Classification criteria alone are not diagnostic and should not be used as checklist. PEST is only intended for patients with PsO, but due to its low specificity more than half of the patients who screen positive do not have PsA. Screening questionnaires can help raise awareness of PsA among patients with PsO.
**Statement** **CR10: Imaging alone cannot diagnose or exclude PsA and must be considered in context.**
Extra-articular manifestations and enthesitis may be difficult to assess clinically. If using imaging, be aware of alternative causes of apparent inflammation in/around the joint, including mechanical tendonitis or OA. If inflammatory axial disease is a concern, MRI may be required. Plain radiography alone cannot confirm or exclude a PsA diagnosis.

**Theme 2: Disease assessment**
**Statements** **CR13: Holistic patient assessment should include an assessment of disease activity, functional impairment, and broader impact from a patient perspective.** **CR14: Routine and regular use of patient-reported outcome measures is recommended.**
PsA has a very broad impact on QoL (which includes pain, fatigue, ability to work, etc.), and there is a need to capture the patient perspective in terms of assessments. Impact on QoL may not only be due to PsA symptoms, but also concomitant conditions, e.g. FM, which need to be identified and managed to determine a treatment approach through shared decision-making. The use of PROMs in PsA has been associated with better self-management, self-efficacy and outcomes. PsAID-12 or a similar tool should be considered as an adjunct for routine monitoring. PsAID-12 responses to individual questions can be more useful for measuring total impact of disease, than a total score. Best practice is both collecting PROMs and using them to facilitate effective communication and shared decision-making. The results of PROMs should be available to patients and physicians. It is good practice to collect and monitor PROMs as a matter of routine (either via a hospital PROMs system or external digital tool). PROMs that are collected should be reflective of the individual patient and of local needs (e.g. linguistically).
**Statement** **CR15: If auditing quality of care, consider including patient-reported experience measures.**
When collecting feedback on patients’ experience, including shared decision-making and goal-setting, tools such as the Commissioning for Quality in Rheumatoid Arthritis Patient-Reported Experience Measure (CQRA PREMS) questionnaire may be useful.
**Statement** **CR16: As a minimum, HCPs caring for someone with PsA should include assessment of joints, enthesitis, spine, skin and comorbidities.**
Assess 66/68-joint count, not just 28-joint count. As a minimum, assess enthesitis using the Leeds Enthesitis Index and also consider other symptomatic areas. Assess inflammatory spinal symptoms and consider appropriate investigations. Assess skin disease activity—consider BSA and refer to dermatology if >3 palms. Encourage all clinicians assessing patients with PsA to ask about high-impact sites (genitals, scalp, nails and natal cleft). No formal assessment is required for comorbidities, but patients should be asked about relevant signs and symptoms. Key comorbidities include metabolic syndrome, diabetes and non-alcoholic fatty liver disease. EULAR CV guidelines include PsA; CV risk is recommended to be assessed every 5 years. Consider using digital tools to collect and monitor patient outcomes.
**Statement** **CR17: Imaging may be used as an adjunct to support clinical decision-making in terms of whether to change/escalate therapy.**
US/MRI can complement clinical assessment of disease activity. Structural changes in the context of PsA can identify patients at risk of progression.

**Theme 3: Comorbidities**
**Statement** **CR18: Given the limited data on the management of many common comorbidities in the PsA population, we recommend using appropriate condition-specific recommendations to guide management of problems such as hyperlipidaemia, hypertension, diabetes, etc.**
Recommended comorbidities to be assessed and managed include: Cardiovascular disease Metabolic syndrome Diabetes Liver disease Chronic infections Bone health FM Reproductive health Mental health Relevant guidance for the management of comorbidities includes the following: NICE obesity guidelines EULAR CV guidelines (which recommend a CV risk assessment for patients with PsA every 5 years) GRAPPA treatment recommendations
**Statement** **CR19: Treatment of comorbidities in patients with PsA should utilize a multidisciplinary team management approach incorporating primary care and appropriate specialists in secondary care.**
It is recommended that rheumatologists support primary care colleagues and liaise closely with other specialities regarding comorbidities. Liaison with other specialities needs to be effective and timely.
**Statement** **CR20: In PsA patients who are overweight/obese, a proactive approach to weight loss should be considered following national guidelines and local services.** **CR21: In PsA patients who are depressed, proactive management should be considered following national guidelines and local services.**
Comorbidities that directly impact the disease include mental health conditions and obesity (*vs* conditions impacting health overall, such as cardiovascular disease). Clinicians should be aware of NICE guidelines for obesity (the treatments and treatment eligibility criteria have been updated). Clinicians should be aware of NICE guidelines for the treatment and management of depression and anxiety. Clinicians should be aware of adverse event profiles and contraindications of pharmacological therapies, and should refer to the SmPC of specific therapies for guidance.
**Statement** **CR22: Be aware that some comorbidities (depression, fatty liver disease) have implications for the pharmacological management of PsA and should be considered before therapy initiation.**
Depression may need to be considered in the context of therapy selection for PsA to avoid potential drug toxicity. Appropriate monitoring is necessary with potentially hepatotoxic PsA disease-modifying drugs.

**Theme 4: Management**
**Statement** **CR25: For guidance on pharmacological management of PsA, refer to national and international treatment recommendations.**
Recommended guidelines include those from BSR, EULAR and GRAPPA. It is useful for rheumatologists to have an awareness of the topical armamentarium for PsO and be familiar with common, effective topical preparations. Refer to NICE guidance for topical treatment recommendations for PsO.
**Statement** **CR26: The use of corticosteroids in PsA should be strictly minimized, with proactive consideration of alternative therapies.**
There is very convincing evidence around the toxicity profile of steroids over long-term use. Even at low doses, long-term use is associated with multiple adverse outcomes and contributes to the burden of comorbidity. There is a role in some patients for i.m. or IA use, but this should be minimized and ideally reserved for those who are already initiated on other biologic or systemic therapies.
**Statement** **CR27: Caution should be exercised in the tapering of steroids in people with PsA, due to the significant risk of PsO flare associated with steroid withdrawal, and patients should be informed of this risk.**
Even in people with mild PsO, the highest risk of skin flare is in patients not on concomitant therapies for their PsO. When there is a need to control active joint disease or inflammation, i.m. or local joint injections may be preferable to oral steroids, because of a lower risk of flare, but be aware that withdrawal may cause a reaction in the skin.
**Statement** **CR28: Smoking cessation support is strongly recommended, in line with current national guidelines.**
The BSR PsA guidelines 2022 provide helpful guidance on this topic. Provide appropriate signposting to encourage patients to quit smoking.
**Statement** **CR29: Patients with PsA should be advised to undertake muscle strengthening and general aerobic exercise. The exercise activity should take into account current disease activity, comorbidities and patient preference.**
There is a lack of evidence to support recommendation of specific types of exercise for specific patient disease phenotypes. There are general benefits of cardio/resistance exercise (MH, fall risk/balance, muscle strength) that may outweigh the risk of worsening symptoms in the presence of musculoskeletal manifestations. HIIT exercise may be beneficial, and showed benefit and no worsening in patients with stable disease.
**Statement** **CR30: Prompt treatment of active inflammation is recommended to improve long-term outcomes. Referral and management within an early inflammatory arthritis clinic is recommended**
Patients with quicker diagnosis and who receive earlier treatment do better across inflammatory arthritides in general. In PsA, the disease phenotype can evolve and worsen over time—early therapy may alter the disease course. There may be underestimation of the extent and severity of subclinical disease (detected by imaging but not examination). Thorough assessment is required, particularly in oligoarticular disease.
**Statements** **CR31: A treat-to-target management strategy is recommended, in line with national** **and international recommendations** **.** **CR32: Target selection should consider all disease manifestations in PsA. Minimal disease activity is the evidence-based multidomain target for treatment in PsA.**
Treat-to-target is recommended by both BSR and EULAR PsA guidelines. Data show that use of a treat-to-target approach can improve clinical outcome, QoL and reduce radiographic damage. Clinics should be set up in a way that facilitates a treat-to-target approach. When considering target selection and measurement: Take the patient’s shoes off and conduct a full 66/68-joint count (not just 28-joint count). The Leeds enthesitis index is quick, easy and PsA specific. MDA is the gold standard. Measure disease activity AND impact (PsAID/PRO).
**Statement** **CR33: There should be shared decision-making and alignment of patient and physician goals when discussing treatment options.**
Any goal should be in the context of the patient’s needs and any risks associated with treatment.
**Statement** **CR34: Collaborative working across key specialities (dermatology, gastroenterology, ophthalmology) is recommended to optimize outcomes for people with PsA; multidisciplinary clinics are recommended.**
A good working practice would include having named contacts within relevant specialities who are available for timely contact for referrals or discussions. There is a need to work with the appropriate colleagues, depending on the patient – individualized care for each individual. Close collaborative working in an MDT clinic can help to upskill rheumatologists in the long term.

BSA: body surface area; BSR: British Society for Rheumatology; CQRA: Commissioning for Quality in Rheumatoid Arthritis; CR: clinical recommendation; CV: cardiovascular; FCP: first contact practitioner; GRAPPA: Group for Research and Assessment of Psoriasis and Psoriatic Arthritis; HCP: health-care professional; HIIT: high-intensity interval training; MDA: minimal disease activity; MDT: multidisciplinary team; MH: mental health; MSK: musculoskeletal; NICE: UK National Institute for Health and Care Excellence; PEST: Psoriasis Epidemiology Screening Tool; PsAID: Psoriatic Arthritis Impact of Disease questionnaire; PREM: Patient Reported Experience Measure; PRO: patient-reported outcome; PROM: patient-reported outcome measure; PsO: psoriasis; QoL: quality of life; SmPC: Summary of Product Characteristics.

### Diagnosis

Within the ‘Diagnosis’ theme (Table 1), the TLR was used to investigate risk factors associated with the development of PsA. Age [8], BMI [9, 10], severity of PsO [10–12] and duration of PsO [13] emerged as strong predictive indicators (in a Danish registry study of 10 011 patients with PsO, the mean duration of PsO at PsA onset was 3.5 years [13]). Despite anecdotal observation of joint stiffness as a predictive indicator in clinical practice, the published evidence remains inconclusive. The SC felt it important to distinguish between true ‘risk factors’, and co-occurring symptoms and features of the underlying disease returned by the TLR such as arthralgia [10] and spondylitis [12]; however, the importance of ensuring that patients with peripheral/axial disease are not ‘missed’ was emphasized. The importance of suspecting PsA in patients with PsO and ≥1 extra-articular manifestations was also highlighted. Similarly, there was overlap between risk of developing PsA and some key comorbidities. The SC agreed that obesity or high BMI should be treated as an independent comorbidity; the same applies to depression [3, 14], with guidance provided for these. Low-quality evidence pertaining to the presence of genetic risk factors was noted, but was beyond this programme’s scope, given its practical focus for clinical use.

Given the heterogeneity of PsA, it is of paramount importance to screen patients with PsO, who represent the main at-risk group [15]. Screening tools available in a primary care setting were investigated, including the German Psoriasis Arthritis Diagnostic (GEPARD) patient questionnaire [16], the Toronto Psoriatic Arthritis Screen II (ToPAS II), the Psoriatic Arthritis Screening and Evaluation (PASE), the Psoriasis Epidemiology Screening Tool (PEST) and the Early Arthritis for Psoriatic Patients (EARP) [17]. PEST was selected as the most practical, user-friendly tool for those managing patients with musculoskeletal conditions in primary care, in alignment with UK National Institute for Health and Care Excellence (NICE) guidelines [18]. While sensitivity of screening tools is generally adequate, their specificity is relatively poor [19]; assessment by a rheumatologist is the gold standard for making a diagnosis of PsA, and the key purpose of screening tools is to prompt consideration of referral to rheumatology services.

Adequate timing for referral from primary to specialist care was also agreed upon, aligning with the recommendations of the National Early Inflammatory Arthritis Audit (NEIAA), which advises 3 weeks [20]. The association between diagnostic delay and poorer outcomes in PsA is well documented [21], with longer time to diagnosis/specialist care linked to a more severe disease course and worse outcomes [22].

### Disease assessment

The recommendations within the ‘Disease Assessment’ theme (Table 2) aim to achieve two key objectives: to highlight the need for individualized assessments addressing factors affecting the individual most significantly, and to provide practical guidance for assessing PsA in the clinic.

PsA has a notably broad impact on quality of life (greater than PsO alone [23]), due to associated symptoms of pain and fatigue, among others, leading to impairments in functional ability and ability to work [3]. This impact may not only be linked to PsA symptoms but also to comorbid conditions, including mental health conditions, which need to be identified and managed as early as possible. Extra-articular manifestations, as previously mentioned, can provide important diagnostic indicators, but are also important to assess on an ongoing basis due to their impact on the burden of disease and as a factor in driving therapy selection [24].

Evidence from the TLR suggested that sex is closely linked with disease course in PsA, resulting in distinct clinical presentations in men and women. Women reported worse quality of life associated with higher levels of disability, fatigue, pain and overall disease severity, as well as a lower likelihood of achieving remission [25]. Men with PsA experienced less overall functional impairment, but a higher impact on their self-esteem [26].

Given the variability in patients’ experience of PsA, it is recommended that the Psoriatic Arthritis Impact of Disease (PsAID-12) questionnaire be used at every consultation. PsAID-12 covers all key domains, and can be administered digitally [27]; it was endorsed at OMERACT2018 as a core outcome measure for assessing PsA-specific health-related quality of life [15]. While recognizing that a complete skin examination at every visit may be challenging in practice, it is an aspirational goal. Special attention should be paid to challenging body areas like the natal cleft, genitals, palmoplantar sites, nails, and scalp, as well as sites prone to enthesitis; tools such as the Leeds Enthesitis Index are easy to administer and provide a comprehensive assessment as a minimum [28]. Evaluation of the patient experience should also be conducted, using a tool such as the Patient Reported Experience Measures tool provided by Commissioning for Quality in Rheumatoid Arthritis [29]. Other assessments advised as part of routine PsA care include cardiovascular risk evaluation, recommended every 5 years, based on EULAR cardiovascular guidelines [30].

Overall, it was clear that while there are minimum quality standards for assessments that form part of day-to-day PsA care, the heterogeneity of the condition requires that the patient perspective be at the centre of the assessment, goal setting and decision-making process; the utility of any outcome measurement tool is dependent on clear communication between the health-care professional and the patient.

### Comorbidities

Recommendations (Table 3) and implications for clinical practice (Table 5) were made for assessment and management of comorbidities, with specific guidance for high-impact conditions, such as depression and obesity.

The SC distinguished between comorbidities that affect a patient’s health overall (such as cardiovascular disease), those that directly impact PsA outcomes (including depression [14], obesity [31] and FM [32]), and those with implications for the treatment of PsA due to contraindications with pharmacological therapies, such as fatty liver disease [33]. Obesity should be addressed for optimal PsA outcomes, using lifestyle and/or treatment interventions. Both NICE obesity guidelines and EULAR cardiovascular guidelines provide useful direction for clinicians [30, 34]. The published literature indicates a positive impact on treatment outcomes in patients with obesity who lose at least 5–10% of their body weight [35]. GRAPPA and EULAR guidelines are other useful resources for clinicians for the management of patients with PsA and depression or obesity [33, 36, 37], while EULAR and the European Society of Cardiology have provided guidance on the management of cardiovascular risk [30, 38]. In addition, comorbidity guidance for PsO may have clinical utility in PsA [39].

The TLR indicated insufficient literature regarding the outcomes of coordinated management of comorbidities in patients with PsA; more evidence is needed. However, extensive experience working within multidisciplinary teams demonstrates that any successful comorbidity management approach requires collaboration with and support from primary care and relevant specialists. It is paramount that clinicians do not consider PsA as a disease existing in a vacuum, and instead address the patient’s health in totality, proactively engaging with them to monitor risk factors and assess potential and existing comorbidities.

### Management

Recommendations (Table 4) and implications for clinical practice (Table 5) within management cover the benefits of early intervention, lifestyle modifications, treating to target and the risks associated with the use of CSs. Guidance on pharmacological therapies is given in extant guidelines and is outside the scope of this work.

Regarding therapy initiation and goal setting, early intervention was agreed to be of paramount importance [4], which may include management in early arthritis clinics [40] and assessment for subclinical enthesitis [41, 42]. Patients with PsA are presenting later and receiving less therapy than patients with RA, and delay in presentation has been associated with poorer outcomes [21, 43]. A thorough early assessment is advised, since in early PsA the extent and severity of disease can be underestimated, particularly in polyarticular disease. It has been observed that the disease phenotype can worsen over time [44]; thus, early therapy may alter the disease course [45] (though data are lacking). Preliminary evidence indicates early biologic treatment of PsO may delay PsA onset [41], although the findings on this are conflicting [46], highlighting the need for additional population-based research.

Lifestyle factors can play a key role in PsA management. Smoking cessation is strongly recommended, in alignment with guidance provided by BSR [1]. There is evidence that exercise is linked to a reduced risk of PsA [31], and that patients with PsA can tolerate high-intensity training without worsening of disease activity [47], despite persisting concerns around mechanical stress triggering an inflammatory response or enthesitis. However, there is a lack of evidence to support the recommendation of specific types of exercise, and given that patients may be unsure what is safe for them, exercise regimens should be tailored to the individual, and their current fitness level and degree of disease activity [48].

For disease activity and therapy monitoring, patient-reported outcome measures (PROMs) were regarded by the SC as useful to include alongside standard clinical assessments. These can be collected digitally, but must reflect the individual and local need in terms of usability, language and health literacy. A treat-to-target model incorporating PROMs of significance to the individual forms the backbone of recommendations in this theme (Table 4).

The use of CSs in PsA management was discussed. In alignment with national and international guidelines, the SC agreed that, while steroids serve a notable role, their use should be minimized in PsA [1, 36, 49, 50]. Treatment with systemic DMARDs prior to introducing steroids may minimize risk of psoriasis skin flares, although supporting data are limited. The committee agreed that oral steroids should not be included in routine PsA management, particularly at high doses (≥10 mg prednisolone daily) or over the long term, though i.m. or local joint injections may be considered in carefully selected cases (alongside other treatments such as DMARDs or biologics) with proper consideration being given to the risk of rebound psoriasis skin flares. The need to communicate these nuances to patients was highlighted; it is important that patients appropriately understand the risk of increased skin disease or erythrodermic reaction. The risk may be higher in patients with unstable skin disease or a previous erythrodermic reaction. The importance of an effective dermatology and rheumatology multidisciplinary approach was highlighted for optimal management; the SC noted that there is room for improvement on this front, and that there is a pressing need to find a balance between treatment of the joints and the skin to maximize patient quality of life.

### Patient votes

Two recommendations did not reach consensus among the patient voters. The first recommendation, within the ‘Comorbidities’ theme, was: ‘In PsA patients who are overweight/obese, a proactive approach to weight loss should be considered following national guidelines and local services’—for which only 60% consensus was achieved. Patient feedback highlighted that this advice is relevant for the whole population and should not serve as a specific feature in PsA recommendations. Moreover, patients felt that currently, patient–health-care professional discussions around weight are not approached in a positive or constructive manner, and thus improvements should be made by clinicians to achieve less negative, more realistic conversations on weight loss.

The second recommendation that did not achieve patient consensus was: ‘Treat to target in PsA recommendations have stated that the target should be remission or inactive disease.’ Patient voters expressed the view that remission or minimal disease activity is not a realistic goal, and that a a more individualized approach is needed. This aligned with SC discussions around the need for a personalized treat-to-target approach, implementing individualized goals; however, overall remission or minimal disease activity is likely to remain the gold standard from a clinical and population guideline perspective.

## Discussion

In this programme, an SC of 12 health-care professionals in the fields of rheumatology, dermatology and primary care convened with the aim of developing an evidence- and consensus-based set of recommendations for the management of PsA in clinical practice to enhance existing guidance. The objective was to define minimum and best quality standards for day-to-day PsA management, complementing and adding value to existing recommendations and guidelines, and providing a set of practical strategies and tools for achieving these quality standard goals to support clinicians. The majority of recommendations (29/34) achieved 90–100% consensus among the faculty.

Unsurprisingly, the topics generating the most challenging discussions were those pertaining to the coordinated management of comorbidities, and the use of steroids in the treatment of PsA and PROMs to measure its impact in routine clinical practice. Though it was unanimously agreed that a well-coordinated, multidisciplinary approach is required, it was also acknowledged that establishing a multidisciplinary approach is challenging in clinical practice; practical strategies such as raising awareness of screening tools in primary care, and rheumatologists spending some time working in an MDT clinic to gain skills in other areas, are proposed. Concerning CSs, although this programme did not aim to make pharmacological therapy recommendations, the SC agreed that their use should be strictly minimized. Regarding the use of PROMs, much consideration was given to how these could be best applied in clinical practice. In the digital age, it is easier than ever to collect PROMs, and thus the SC agreed these can and should be used in routine practice. However, it was suggested that, in order to be useful, the specific PROMs and collection platform employed must be appropriate and individualized to the patient’s disease state and degree of digital and health literacy, as well as to the local need. The SC also discussed the possibility of linking PROMs to an individualized treat-to-target approach, reflecting an overall theme—PsA is a heterogeneous and multifaceted condition that does not exist in a vacuum, and each patient needs to be considered individually and holistically.

Both the SC and EF were UK based; this may limit the ease of generalizing some of the recommendations to all health-care settings. The limited sample size of the EF, especially among patients, is another limitation; owing to the low number of patients recruited for voting, the results could be easily skewed. Moreover, there was a low degree of engagement from the EF; of the 79 members invited, only 16 voted on the recommendations. Other limitations pertained to the programme’s remit. Pharmacoeconomic and treatment access considerations, and further guidance on identifying and managing extra-articular manifestations, were outside the scope of this work, although the SC acknowledge their significance in holistic patient care. Reproductive health is a key concern for patients with PsA not covered here; BSR guidelines provide comprehensive guidance on pregnancy and breastfeeding [51], but further work is needed.

The two recommendations that did not achieve consensus among patient voters pertained to the management of obesity and using remission or minimal disease activity as a treatment target. However, the patient board provided a rationale for rating recommendations at 6 or less, and in both cases the SC agreed a more targeted and individualized approach is essential for successfully managing comorbidities, such as obesity, and for implementing a treat-to-target approach.

This consensus programme identified critical areas beyond pharmacological therapy where the existing guidance on PsA management could be enhanced. The recommendations and implications for clinical practice aim to provide relevance to health-care professionals and a clinical resource to support the care of patients with PsA. Owing to the practical and specific nature of the recommendations, it is hoped that the guidance can be easily and rapidly implemented into practice for use in conjunction with current guidelines.

## Supplementary Material

keae172_Supplementary_Data

## Data Availability

The data underlying this article is available upon reasonable request to the corresponding author.
