# Supplementary material for: Enhancing current guidance for psoriatic arthritis and its comorbidities: recommendations from an expert consensus panel
Source: Rheumatology (Oxford). 2024 Mar 15;64(2):561–73. doi: 10.1093/rheumatology/keae172 (PMC11781585; doi:10.1093/rheumatology/keae172)
Supplement: keae172_Supplementary_Data [file keae172_supplementary_data.docx]

**Enhancing current guidance for psoriatic arthritis and its comorbidities: recommendations from an expert consensus panel**

**Supplementary appendix**

**Supplementary Figure S1: Modified Delphi process for achieving consensus**

Discuss the results of the consensus voting and dissemination channels

Finalise the draft recommendation wording

Vote on the draft recommendations (SC and EF)

Review and refine the draft consensus recommendations

Approve the question wording, conduct a TLR
and draft the recommendations

Identify the consensus themes and draft questions

EF, extended faculty; SC, steering committee; TLR, targeted literature review.

**Supplementary
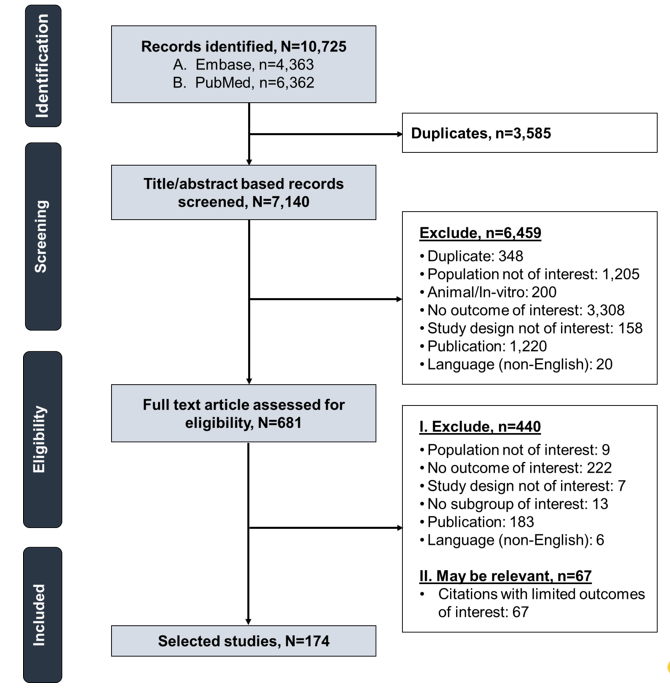
Figure S2: TLR methodology**

TLR, targeted literature review.
